# Genetic Algorithm-Optimized CNN-BiLSTM Framework for Predicting the Remaining Useful Life of IGBT Modules

**DOI:** 10.3390/s26061964

**Published:** 2026-03-21

**Authors:** Yukai Hao, Jiao Wu, Zhiheng Zhang, Yuanhao Wang, Tao Wang, Yujie Liang

**Affiliations:** 1 School of Computer Science and Technology, Xidian University, Xi’an 710126, China; ykhao@stu.xidian.edu.cn; 2 Aeronautics Computing Technology Research Institute, Aviation Industry Corporation of China, Ltd., Xi’an 710068, China; wjiao@mail.nwpu.edu.cn (J.W.); zzhdllgdx@163.com (Z.Z.); 3 Guangzhou Institute of Technology, Xidian University, Guangzhou 510555, China; 17754414529@163.com (T.W.); 24181214073@stu.xidian.edu.cn (Y.L.)

**Keywords:** IGBT, remaining useful life, convolutional neural network, long short-term memory network, genetic algorithm

## Abstract

To address the aging and failure issues that arise during the long-term operation of insulated gate bipolar transistors (IGBTs), this paper proposes a method for predicting their remaining useful life (RUL). The proposed method utilizes a genetic algorithm to optimize a hybrid model that combines a convolutional neural network (CNN) with a bidirectional long short-term memory (BiLSTM) network. First, based on the failure mechanism of IGBTs, various commonly used RUL prediction methods are analyzed and compared. Considering that CNNs are particularly effective at extracting spatial features, while LSTMs excel at capturing long-term dependencies in time-series data, a hybrid CNN-BiLSTM model is developed for RUL prediction, with hyperparameters, including the initial learning rate, optimized using a genetic algorithm. Experimental results demonstrate that the proposed CNN-BiLSTM model achieves superior performance across all metrics compared with benchmark algorithms, and the genetic algorithm significantly accelerates the parameter optimization process and enhances the overall training efficiency.

## 1. Introduction

The Insulated Gate Bipolar Transistor (IGBT), as a mainstream high-power semiconductor device, is widely used in high-voltage and high-power energy conversion applications such as aerospace, renewable energy generation, rail transit, and electric vehicles. In addition, IGBTs play an important role in sensor and sensor network applications. As key components in sensor node power management units, they enable efficient power conversion and regulation for data acquisition, processing, and wireless communication modules. In intelligent sensor systems, IGBTs also provide reliable power switching and amplification for actuator driving and analog signal processing, ensuring stable operation and efficient coordination of edge devices [1]. It plays a vital role in improving energy conversion efficiency while contributing to energy conservation and environmental protection [2]. In particular, aircraft power converters are critical components of onboard power systems, responsible for DC/AC power conversion within the aircraft secondary power system. Currently, IGBT modules are commonly employed as power devices in aviation inverters [3].

An IGBT consists of an N-substrate, P-layer, N+ collector region, and a gate structure, enabling fast switching speed and low conduction voltage drop. For high-power applications, IGBTs are typically packaged in modular form, integrating multiple chips and heat-sinking structures to enhance electrical and thermal performance. However, under actual operating conditions, IGBT modules are exposed to complex environments and highly variable working conditions. They are subjected to long-term electro-thermal coupled stresses and cyclic loads, which can easily induce degradation mechanisms such as solder layer fatigue and bond-wire failure. The aging and failure of IGBTs are mainly caused by the combined effects of thermal, voltage, current, and mechanical stresses, as well as environmental factors. For instance, prolonged high-power operation raises the junction temperature, accelerating material degradation [4,5]. Repeated voltage switching may damage the gate oxide layer or cause localized breakdown, while overcurrent conditions increase power dissipation and lead to excessive device temperatures. As aging progresses, the physical structure of the IGBT gradually deteriorates, resulting in time-dependent variations in key electrical and thermal parameters. If degradation states cannot be identified in a timely manner and failure time cannot be accurately predicted, sudden failures may occur, leading to system shutdowns, equipment damage, or even serious safety incidents. Therefore, it is necessary to focus on IGBT modules used in aviation inverters and investigate their Remaining Useful Life (RUL) [6,7]. Accurate modeling of degradation mechanisms and RUL prediction enables condition awareness and predictive maintenance, avoiding both excessive and delayed maintenance. This improves system reliability and availability, reduces operation and maintenance costs, and provides a scientific basis for ensuring safe operation and lifetime optimization of power electronic systems. In addition, it helps maintenance personnel determine optimal maintenance timing, thereby reducing repair costs and further enhancing system operational reliability [8].

There are many efforts done for RUL prediction. The authors propose the GRU-Augmented Time-Frequency Estimator (GATE) in [9]. The GATE method integrates an autoregressive time-series prediction framework, trained using the Teacher Forcing strategy, allowing it to recursively decode electrical parameters indicative of IGBT aging states from comprehensive time-frequency features. An improved Wiener process method is introduced in [10] to mitigate noise interference and uncertainty in the RUL prediction of high-power IGBT modules. By incorporating the Ornstein-Uhlenbeck process to more accurately model the degradation process, alongside an LSTM network to augment the dataset, the method effectively reduces the uncertainty in RUL predictions. A hybrid RUL prediction approach in provided in [11] to address the uncertainty and data insufficiency challenges in the RUL prediction of high-speed train IGBT modules. This method combines Variational Mode Decomposition, Kernel Density Estimation-Bidirectional Long Short-Term Memory (KDE-BiLSTM), Wiener process modeling, and other techniques, successfully handling issues related to multivariance, uncertainty, and insufficient performance degradation data. A novel online monitoring and prediction method is presented in [6], which combines Principal Component Analysis (PCA) and Feedforward Neural Network (FFNN) techniques. This approach extracts relevant information through time-domain analysis and performs online regression of trend parameters using PCA and FFNN. A hybrid CNN and Transformer model is provided in [2]. By using accelerated aging power cycling tests and the variation of saturated voltage drop as key aging indicators, combined with data preprocessing and model training, the prediction accuracy is significantly improved. A novel RUL prediction method based on the Snake Optimizer (SO) in [12] to optimize the Bi-LSTM neural network. The method employs data preprocessing, an attention mechanism, and hyperparameter optimization to significantly enhance the model’s prediction accuracy. Deng et al. [13] further combined the sequence decomposition algorithm with the BiLSTM network, reduced the accumulation of prediction errors for non-stationary degradation signals, and improved the adaptability of the model in industrial noise environments.

Different from the work above, this paper presents a RUL prediction framework based on an optimized deep learning model. First, the degradation mechanisms of IGBTs are investigated, and several representative data-driven RUL prediction approaches are systematically reviewed and compared to clarify their respective advantages and limitations. Second, a hybrid CNN-BiLSTM prediction model is constructed, which leverages the strong spatial feature extraction capability of convolutional neural networks and the effectiveness of bidirectional long short-term memory networks in modeling long-term temporal dependencies in degradation time-series data. To further enhance model performance, a genetic algorithm is employed to automatically optimize key hyperparameters, thereby improving convergence behavior and training efficiency.

The main contributions of this paper are summarized as follows:

(1) A hybrid CNN-BiLSTM algorithm is proposed for IGBT remaining useful life prediction, combining convolutional neural networks for effective feature extraction with bidirectional long short-term memory networks for capturing temporal degradation characteristics.
(2) A genetic algorithm is introduced to adaptively optimize key hyperparameters of the proposed model, leading to improved convergence performance and enhanced training efficiency.
(3) The effectiveness of the proposed approach is verified using an IGBT aging dataset, where extensive experimental results demonstrate the superior prediction accuracy and robustness of the optimized CNN-BiLSTM model compared with existing methods.

## 2. Preliminaries

### 2.1. Main Methods for Predicting the RUL of IGBTs

From the perspective of prediction mechanisms, existing approaches for estimating the remaining RUL of IGBT modules can be broadly classified into two categories: model-based methods and data-driven methods. Model-based approaches include analytical methods and physics-based models that rely on explicit degradation mechanisms, while data-driven approaches mainly consist of machine learning-based techniques and statistical time-series methods that infer degradation patterns directly from historical data.

#### 2.1.1. Model-Based Methods

Analytical-based methods refer to empirical relational models that establish correlations between the number of failure cycles and degradation-related physical quantities using accelerated test data. These methods do not explicitly consider the internal physical failure mechanisms of IGBT modules during the modeling process. Common physical quantities employed in analytical models for IGBT modules include load current, cycle frequency, maximum temperature, temperature fluctuation amplitude, temperature rise rate, wire diameter, and stress parameters applied during accelerated life testing. For example, the Coffin–Manson and Lesit models primarily consider junction temperature, whereas the Norris–Landzberg and Bayerer models further incorporate additional factors such as cycle frequency, heating time, and load current [14,15]. Analytical models are typically developed by fitting failure data obtained from accelerated aging experiments, in which stress levels are intentionally increased to induce failures within a shorter time frame while preserving the underlying failure mechanisms.

Physics-based RUL prediction models are developed within the physics-of-failure (PoF) reliability framework. For IGBT modules, these models mainly focus on packaging-related failures that evolve gradually and are characterized by changes in key structural or material parameters, such as those associated with solder layers and bonding wires. Models for solder layer degradation are typically formulated based on plastic strain, creep strain, energy dissipation, and cumulative damage, whereas bonding wire degradation models commonly rely on strain, stress, and fracture mechanics principles [16]. Accurate RUL prediction using physics-based models depends on the correct identification of the dominant failure mechanisms and their locations, which is essential for selecting an appropriate degradation model.

#### 2.1.2. Data-Driven Methods

Machine learning-based methods utilize data processing techniques, such as neural networks, to train on operational data and learn the mapping between observed data and the remaining useful life of IGBT modules. The trained models are then employed to predict the RUL of in-service IGBT modules. This modeling approach is relatively straightforward and does not require explicit modeling of the physical relationships between degradation data and failure mechanisms [17]. However, data-driven RUL prediction methods for IGBT modules generally require sufficient and reliable datasets to achieve high prediction accuracy, which strongly depends on the availability of failure data. Although increasing the amount of failure data can improve prediction performance, acquiring such data is often difficult and costly in practice [18]. To enhance the accuracy and applicability of machine learning-based RUL prediction methods, it is essential to first improve data acquisition techniques to obtain more representative failure data. Additionally, optimizing individual algorithms or integrating multiple machine learning methods can further improve prediction accuracy while reducing reliance on large-scale datasets.

Statistical sequence-based methods rely on historical operational data of IGBT modules to construct statistical models, such as fault probability density functions (PDFs). These PDFs are obtained through statistical analysis of historical data, using commonly adopted techniques such as Monte Carlo simulation and particle filtering [19]. When constructing an explicit RUL prediction model for IGBT modules is infeasible or excessively complex, machine learning-based and statistical sequence-based methods are often employed as effective alternatives.

### 2.2. Main Neural Networks for Predicting the RUL of IGBTs

#### 2.2.1. Convolutional Neural Network

**Figure 1:**
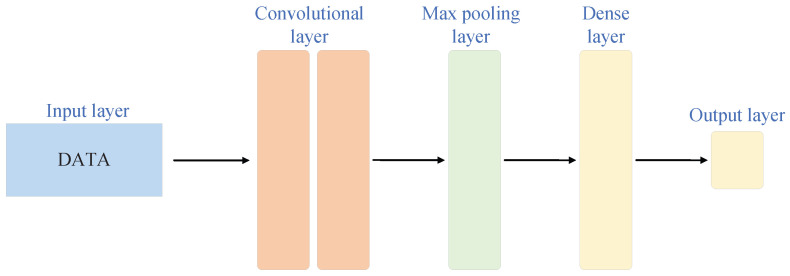
Simple CNN architecture.

A convolutional neural network (CNN) is a type of deep learning model, as shown in Figure 1. It is primarily used for processing two-dimensional or three-dimensional data, such as images and videos. The core function of a CNN is to extract spatial features from input data using local receptive fields and weight-sharing mechanisms. Compared with fully connected networks, a CNN replaces fully connected operations in traditional networks with convolutional layers, significantly reducing the number of parameters and improving computational efficiency. Structurally, a typical CNN commonly consists of multiple layers, including convolutional layers, pooling layers, activation functions, and fully connected layers [20]. Convolutional layers apply different filters to the input data for localized nonlinear transformations, capturing spatial features at various scales and positions. Pooling layers reduce computational complexity and enhance model robustness by down-sampling local regions of the feature maps. Finally, after multiple layers of convolution and pooling, a CNN converts high-dimensional feature maps into low-dimensional vectors and performs classification or regression tasks through fully connected layers and activation functions.

#### 2.2.2. Recurrent Neural Network

**Figure 2:**
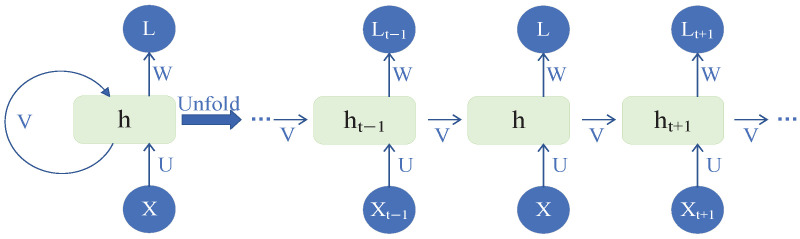
The schematic diagram of RNN architecture.

Recurrent neural network (RNN), a type of neural network, is primarily used for processing sequential data. An RNN propagates intermediate results across different time steps, allowing each prediction to depend not only on the current input but also on the intermediate results from all previous time steps. The structural schematic diagram of RNN is shown in Figure 2.

As shown in Figure 1, $X_{t}$ is the input at time *t*, *W* is the weight parameter of the hidden state, and $h_{t}$ at time *t* that depends on $X_{t}$ and the hidden state. The internal memory $h_{t}$ is computed using Equation (1), where g() represents the activation function (typically Tanh), *U* and *W* are adjustable weight matrices for the hidden state (h), *b* is the bias, and *X* denotes the input vector. Although the memory capability of an RNN allows it to effectively handle temporal sequences, it can only model relatively short sequences and is prone to gradient explosion and vanishing gradient problems. Therefore, in practical engineering applications, variants of RNN are commonly employed.

(1) $$\begin{array}{c} h_{t}=g\left(WX_{t}+Uh_{t}+b\right) \end{array}$$

#### 2.2.3. LSTM Network

**Figure 3:**
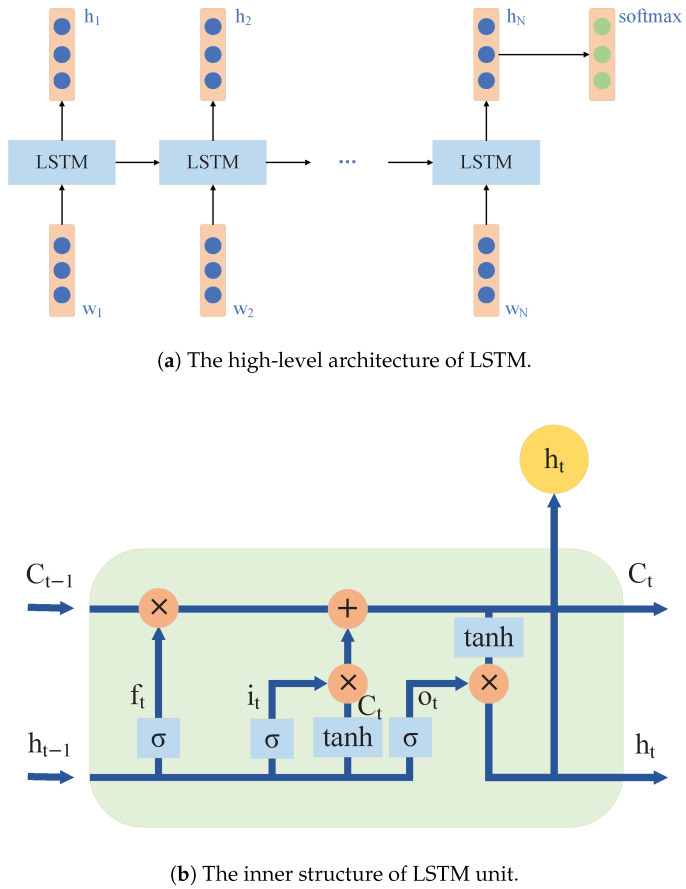
The structural schematic diagram of LSTM.

A long short-term memory network (LSTM) is a variant of RNN that introduces linear connections in the hidden layer state A and incorporates three types of gating units, i.e., the input gate, the forget gate, and the output gate. These operations allow the feature information of input samples to be selectively propagated backward, thereby addressing the problems of vanishing gradients and the decreasing sensitivity of later time steps to earlier ones in the RNN [21]. Figure 3 shows the structural schematic diagram of an LSTM.

The process by which an LSTM controlling the flow of information through gated units is as follows: (1) the forget gate $f_{t}$ determines the proportion of historical information to retain (between 0 and 1), as shown in Equation (2); (2) the input gate $i_{t}$ determines the proportion of input information, as shown in Equation (3); (3) the forget gate $f_{t}$ and input gate $i_{t}$ together control how much of the historical information $C_{t-1}$ to forget and how much new information ${\tilde{C}}_{t}$ to add, thereby updating the long-term memory cell $C_{t}$, as shown in Equations (4) and (5); (4) the output gate $o_{t}$ determines how much information from the long-term memory cell $C_{t}$ is output to the short-term memory cell $h_{t}$, as shown in Equations (6) and (7). Here, $X_{t}$ is the input at time *t*; $W_{f}$, $W_{i}$, $W_{c}$ are the parameter matrices from the input layer to the hidden layer; $U_{f}$, $U_{i}$, $U_{c}$ are the recurrent parameter matrices from the hidden layer to itself; and *b* is the bias parameter matrix. The sigmoid activation function ensures that the outputs of the three gates are between 0 and 1. The value of the long-term memory cell $C_{t}$ changes slowly and primarily remembers information from previous time steps. The value of the short-term memory cell $h_{t}$ changes rapidly and mainly remembers new input information. They jointly represent the cell state at time *t*. This naturally characteristic of LSTM makes it well-suited for predicting highly correlated temporal sequences.

(2) $$\begin{array}{c} f_{t}=\sigma \left(W_{f}X_{t}+U_{f}h_{t-1}+b_{f}\right) \end{array}$$

(3) $$\begin{array}{c} i_{t}=\sigma \left(W_{i}X_{t}+U_{i}h_{t-1}+b_{i}\right) \end{array}$$

(4) $$\begin{array}{c} {\tilde{C}}_{t}=tanh\left(W_{c}X_{c}+U_{c}h_{t-1}+b_{c}\right) \end{array}$$

(5) $$\begin{array}{c} C_{t}=f_{t}*C_{t-1}+i_{t}*{\tilde{C}}_{t} \end{array}$$

(6) $$\begin{array}{c} o_{t}=\sigma \left(W_{o}X_{t}+U_{o}h_{t-1}+b_{o}\right) \end{array}$$

(7) $$\begin{array}{c} h_{t}=o_{t}*tanh\left(C_{t}\right) \end{array}$$

#### 2.2.4. Bi-Directional LSTM Network

**Figure 4:**
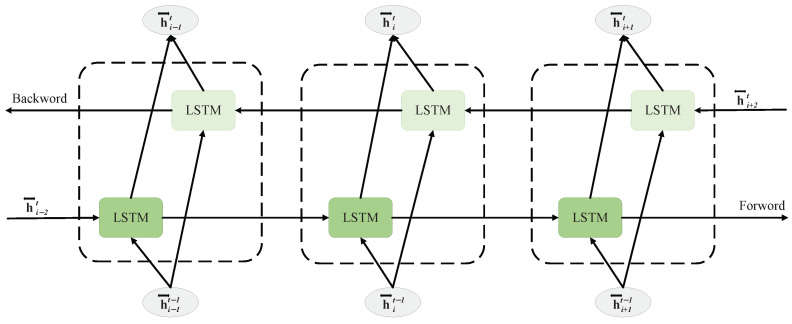
The architecture of a BiLSTM model.

To enhance the capability of capturing temporal sequence information, the bidirectional long short-term memory network (BiLSTM) is used which is shown in Figure 4. BiLSTM is developed on the basic of LSTM, effectively overcoming the limitation of LSTM, which can only transmit information forward in time [22]. The core design idea of BiLSTM is to construct two parallel LSTM structures: one is the “forward LSTM”, which processes data from the beginning (*t* = 1) to the end (*t* = T) along the time axis, focusing on capturing historical temporal information; the other is the “backward LSTM”, which operates in reverse from the end (*t* = T) to the beginning (*t* = 1) along the time axis, capturing future temporal correlations. These two LSTMs share the same input sequence but independently update their cell states and gating units. At each time step *t*, the short-term memory outputs of the forward and backward LSTMs are fused through concatenation or other appropriate operations to form the final hidden state at that moment. This bidirectional structure allows the BiLSTM to utilize the complete temporal context of past, present, and future at each time step, making it particularly suitable for tasks that require integrating information from both past and future.

## 3. Predicting the RUL of IGBT Based on Genetic Algorithm Optimized CNN-BiLSTM

Given that CNNs are well suited for learning representative spatial features, while LSTMs are capable of modeling long-range temporal dependencies in sequential data, a CNN-BiLSTM hybrid architecture is constructed for RUL estimation in this section. Furthermore, key hyperparameters of the model are automatically tuned through the genetic algorithm to enhance prediction performance.

### 3.1. CNN-BiLSTM Network

The method of hybridizing and integrating neural network models demonstrates significant advantages in various application scenarios. By combining CNN with BiLSTM, this method not only fully leverages the strengths of CNN in extracting spatial features but also exploits BiLSTM’s ability to effectively capture long-term dependencies in time-series. On the one hand, this combination achieves efficient extraction of spatiotemporal features; on the other hand, it provides strong support for multimodal data processing.

The CNN-BiLSTM combined model consists of input layers, CNN layers, BiLSTM layers, fully connected layers, output layers. Its model configurations are summarized in Table 1. CNN here refers to the one-dimensional convolutional neural network (1D-CNN), which is specifically designed for sequential data. The convolutional layer consists of multiple convolutional kernels, which extract local features from the input data. Each convolutional kernel detects a specific type of pattern or edge. By stacking kernels of different sizes and quantities, the model can learn hierarchical abstract features. The pooling layer reduces the spatial dimensions of the feature maps and decreases computational load. Max pooling is commonly used to retain the maximum values in local regions, thereby emphasizing prominent features. The 1D-CNN layer is used to extract local time-dependent features that reflect the device degradation state from the input one-dimensional time-series. The BiLSTM layer processes the temporal features extracted by the CNN. It uses gating mechanisms (forget gate, input gate, and output gate) to control the flow of information, enabling effective capture of long-term dependencies in time-series data, making it suitable for data exhibiting temporal correlations. The fully connected layer maps the output of the BiLSTM to the final predictions or classification labels and incorporates activation functions (e.g., ReLU) and dropout layers to prevent overfitting and enhance model generalization.

### 3.2. Hyperparameter Optimization

During model training, hyperparameters have a significant impact on the model’s predictive accuracy, especially for predictions at points with high data variability. The hyperparameters of the CNN-BiLSTM mainly include the number, size, and stride of convolutional kernels; the type and size of pooling layers; and the number of LSTM hidden units, learning rate, batch size, dropout rate, and optimizer. These parameters affect the feature extraction and sequence modeling capabilities, respectively, influencing the model’s accuracy and training efficiency. The number of convolutional kernels determines the model’s sensitivity to spatial features. Pooling operations are used to reduce computational complexity while preserving key features. The number of LSTM hidden units directly affects the sequence modeling capacity. Learning rate and batch size control the convergence speed and stability of the training process, and dropout is used to prevent overfitting. Hyperparameters obtained through trial and error cannot ensure that the CNN-BiLSTM model operates at its optimal performance. To improve its performance in sequence data processing tasks, hyperparameter optimization is required. Common methods for hyperparameter optimization in models include grid search, random search, Bayesian optimization, automated machine learning (AutoML), and swarm intelligence algorithms. The advantages and disadvantages of these methods are summarized in Table 2. After comprehensive consideration, in this paper, a typical swarm intelligence algorithm, namely, genetic algorithm, is selected to optimize the model’s hyperparameters.

### 3.3. GA-CNN-BiLSTM-Based Prediction

The genetic algorithm (GA) is an optimization algorithm based on the theory of biological evolution. Its core idea is to gradually evolve better solutions by simulating the process of population evolution. GA transforms the problem to be optimized into a population-based representation, where each individual represents a possible solution (chromosome). The algorithm iteratively improves the overall fitness of the population through selection, crossover, and mutation operations, eventually approaching the global optimum. GA is suitable for solving complex combinatorial optimization problems, especially when traditional mathematical methods fail to find the global optimum. Typical applications include problems such as the traveling salesman problem (TSP), function optimization, and parameter tuning in machine learning. Its advantage includes the ability to handle nonlinear, multi-peak search spaces and a strong global search capability. The main processes of GA include:

(1) Initialize population. A set of initial solutions (individuals) is randomly generated, each represented by a binary string or other encoding methods.
(2) Calculate fitness. The fitness value of each individual is evaluated according to a predefined objective function. The fitness value is taken as the average of the root mean square error (RMSE) obtained from three repeated training runs. Accordingly, the fitness function can be formally defined as: Fitness = Average(${\mathrm{RMSE}}_{1}$, ${\mathrm{RMSE}}_{2}$, ${\mathrm{RMSE}}_{3}$). The optimization objective of the GA is to minimize this fitness value.
(3) Select parents. Superior individuals are selected as parents based on fitness values using methods such as roulette wheel selection or tournament selection.
(4) Crossover. The genes of the parents are recombined with a certain probability (e.g., single-point or two-point crossover) to generate new individuals.
(5) Mutation. Random mutations are applied to the newly generated individuals to increase population diversity.
(6) Update the population. Some individuals in the original population are replaced by the newly generated ones to form a new generation.
(7) Check termination conditions. If termination conditions such as fitness convergence, number of iterations, or time limit are met, the algorithm terminates; otherwise, the above steps are repeated.

**Figure 5:**
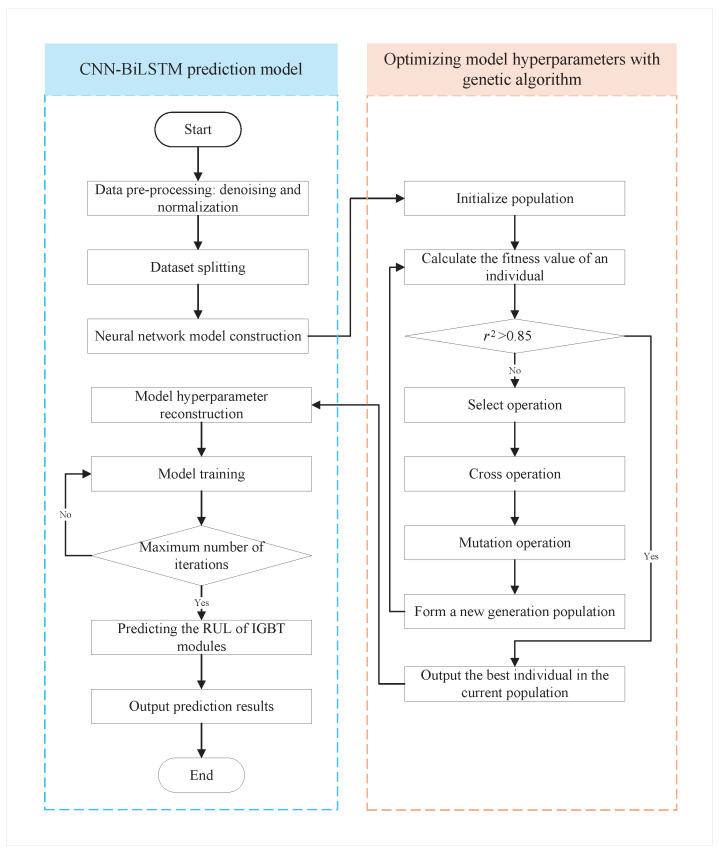
The flowchart for predicting the RUL of IGBT modules based on the GA-CNN-BiLSTM.

In this paper, the genetic algorithm is used to optimize 4 key hyperparameters in the CNN-BiLSTM model: number of convolution kernels, initial learning rate, regularization coefficient and number of LSTM hidden neurons. The flowchart for predicting the RUL of IGBT modules using the GA-CNN-BiLSTM is shown in Figure 5. The GA-CNN-BiLSTM model, which adopts an end-to-end training approach, can automatically adjust model parameters to optimize performance, thereby reducing dependence on manual feature engineering. dependence on manual feature engineering.

The main steps of the RUL prediction model for IGBT based on GA-CNN-BiLSTM are as follows:

(1) Select the feature data and perform preprocessing such as denoising and normalization, and then divide the data into training and testing sets.
(2) Build a CNN-BiLSTM model that integrates CNN and BiLSTM, and initialize the hyperparameters of the CNN-BiLSTM model, including the initial learning rate, number of epochs, batch size, learning decay rate, and decay cycle. Meanwhile, establish the initial population of the genetic algorithm.
(3) Use the average RMSE obtained from multiple CNN-BiLSTM training runs as the fitness value, and optimize the model hyperparameters using the genetic algorithm.
(4) Input the optimal parameters obtained by the genetic algorithm into the CNN-BiLSTM model for training, and predict the remaining useful life of IGBT modules in the testing set to demonstrate the performance of the trained model.

## 4. Performance Evaluation

### 4.1. Experimental Setup

This paper is based on the accelerated aging test data of IGBTs obtained from the NASA PCoE laboratory for model training and validation. The test subject is the IRG4BC30K IGBT chip manufactured by International Rectifier. The experimental data are obtained from a multi-sensor monitoring network deployed on the accelerated aging test platform. Such a sensor network acts as the physical basis for online condition monitoring and predictive maintenance. The quality and integrity of the data acquired by the sensor network directly determine the prediction performance of the subsequent data-driven models [23]. The experiment accelerates the aging process of the device by controlling the package temperature beyond the rated temperature range of the 329–333 °C. During this process, multiple parameters were monitored, including collector current, collector voltage, gate voltage, and package temperature. The configuration of the device consists of connecting the emitter to the ground of the power supply and the collector connected in series with a resistor to the positive terminal of the power supply. The gate is driven by a high-speed amplifier, which amplifies the output of a function generator.

The output signal is smoothed using a bidirectional moving average filter based on an *N*-point sliding window, aiming to suppress noise while avoiding phase shift issues caused by unidirectional filtering. Here, *h* is the equally weighted filter coefficient, as shown in Equation (8), where *N* is length. In practice, *N* is set to 3, meaning a 3-point window filter. y(n) is the bidirectional filter output, as shown in Equation (9), where x(n) is the original time series data. The signal is first filtered in the forward direction, then reversed and filtered again, and finally reversed back and restored. This approach achieves smooth noise reduction with zero phase offset, with the boundaries processed via symmetric extension.

(8) $$\begin{array}{c} h={\left[\frac{1}{N},\frac{1}{N},\ldots ,\frac{1}{N}\right]}^{T} \end{array}$$

(9) $$\begin{array}{c} y(n)=reverse\{filter(h,1,reverse\{filter(hh,1,x(n))\})\} \end{array}$$

**Figure 6:**
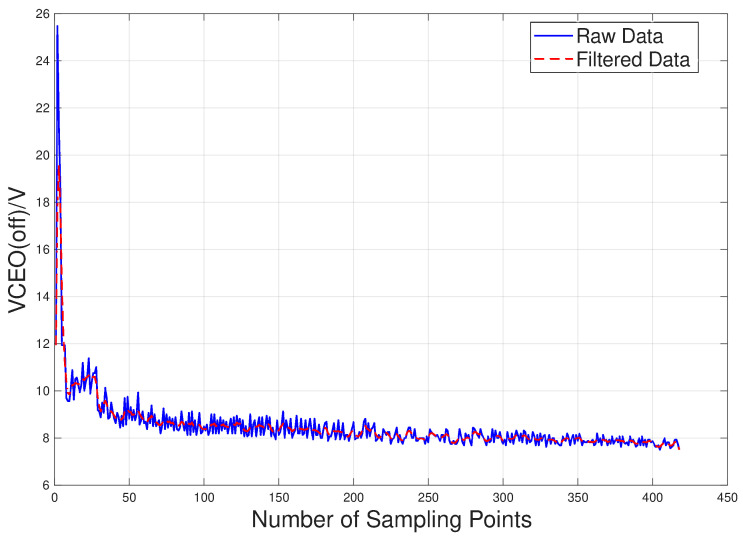
Curve of collector–emitter turn-off peak voltage variation.

During the experiment, a square wave signal with an amplitude of 10 V, a frequency of 10 kHz, and a duty cycle of 40% was applied to the gate of the IGBT device for 172 min. A total of 418 data sets were collected, each containing 100,000 sampling points. A strong indicator was observed when viewing the collector-emitter voltage turn off characteristics. The transient turn-off peak decreased significantly, as a function of both temperature increases and thermal overstress degradation. As a precursor signal for device degradation under thermal overstress, its decreasing trend is correlated with aging time and severity, and can be effectively used for subsequent degradation analysis and prognosis. The raw data and filtered data are shown in Figure 6. The first 80% of the data is used as the training set, and the remaining 20% of the data is used as the test set.

### 4.2. Evaluation Models

To evaluate the performance of the CNN-BiLSTM prediction model in this paper, this model is qualitatively and quantitatively analyzed in comparison with existing models. The models used in the validation process are as follows:

(1) LSTM model. The LSTM-based voltage prediction model considered in this paper.
(2) BiLSTM model. A network model consisting of forward and backward LSTM layers, which can more comprehensively capture the bidirectional dependencies inherent in voltage sequences.
(3) CNN-BiLSTM model. A novel prediction model proposed in this paper that leverages the spatial feature extraction capability of CNNs to capture local dependencies in the input sequence and then feeds these high-level features into a BiLSTM, enabling more accurate modeling of complex temporal dynamics.
(4) GA-CNN-BiLSTM model. A genetic algorithm is introduced during the training of the CNN-BiLSTM model to accelerate the optimization of the network parameters.
(5) TCN-BiLSTM model. The temporal convolutional network (TCN) is employed to capture the dependencies between preceding and following contexts of voltage sequences, thereby further enhancing the model’s understanding and representation of contextual semantics.

The parameters of the CNN-BiLSTM model are summarized in Table 3.

### 4.3. Evaluation Metrics

During the training and evaluation process of machine learning regression models, model performance is usually assessed using mean absolute error (MAE), mean squared error (MSE), root mean squared error (RMSE), and R-squared coefficient of determination ($r^{2}$), as shown in Equations (10)–(13). MAE is the average of the absolute differences between predicted and actual values. It measures the true average level of prediction errors, where a lower MAE indicates better model performance. MSE is the mean of the squared differences between the predicted and the actual values. It is a key metric for evaluating a model’s sensitivity to outliers and prediction errors, where a lower MSE indicates better model performance. RMSE provides an intuitive measure of prediction error magnitude, where a lower RMSE indicates better model performance. $r^{2}$ measures the goodness of fit of a model, indicating the proportion of variance explained by the model, where a higher $r^{2}$ indicates better model performance.

(10) $$\begin{array}{c} \mathrm{MAE}=\frac{1}{m}\sum_{1}^{m}\left|y_{i}-{\hat{y}}_{i}\right| \end{array}$$

(11) $$\begin{array}{c} \mathrm{MSE}=\frac{1}{m}\sum_{i=1}^{m}{\left(y_{i}-{\hat{y}}_{i}\right)}^{2} \end{array}$$

(12) $$\begin{array}{c} \mathrm{RMSE}=\sqrt{\frac{1}{m}\sum_{i=1}^{m}{\left(y_{i}-{\hat{y}}_{i}\right)}^{2}} \end{array}$$

(13) $$\begin{array}{c} r^{2}=1-\frac{\sum_{i=1}^{m}{\left(y_{i}-{\hat{y}}_{i}\right)}^{2}}{\sum_{i=1}^{m}{\left(y_{i}-\bar{y}\right)}^{2}} \end{array}$$

where *m* is the number of samples, $y_{i}$ is the true RUL of the *i*-th sample, ${\hat{y}}_{i}$ is the predicted RUL of the *i*-th sample, and $\bar{y}$ the mean RUL of samples.

### 4.4. Experimental Results and Analysis

The parameters associated with neural network training are summarized in Table 4.

**Figure 7:**
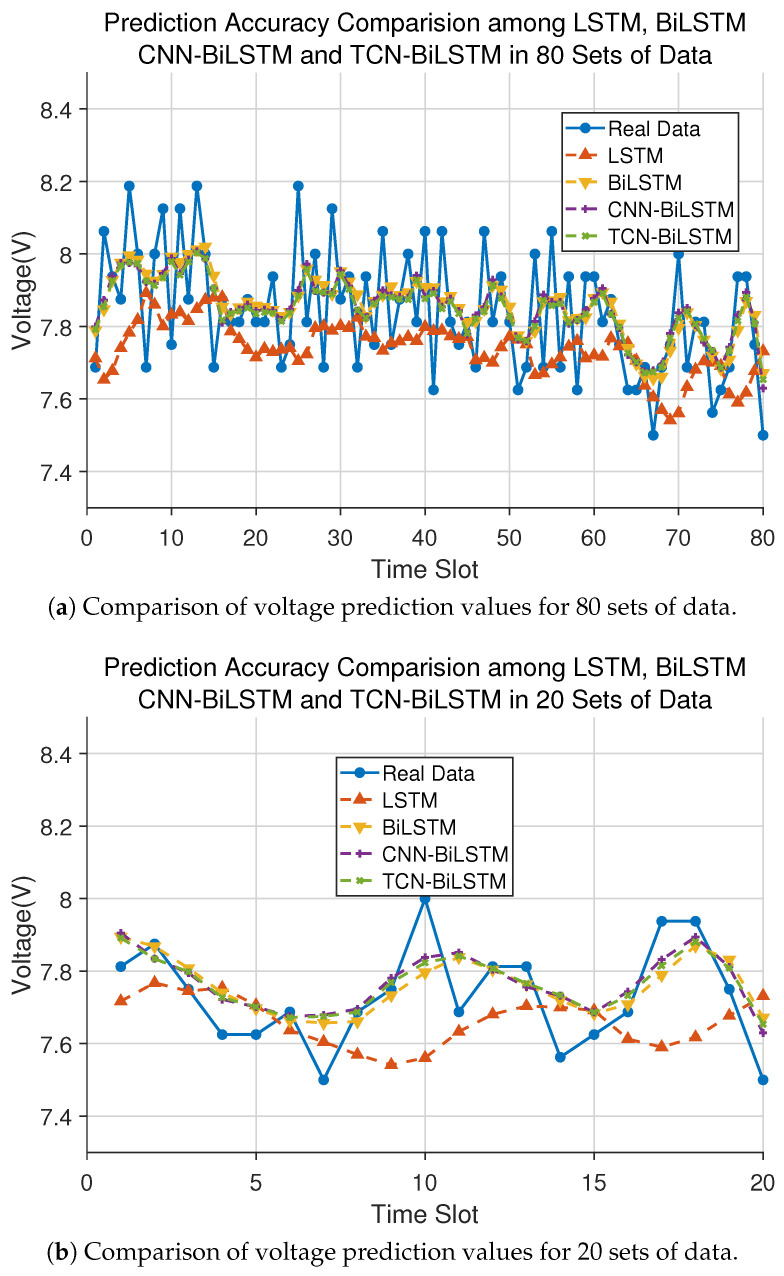
Comparison between IGBT true and predicted values obtained using LSTM, BiLSTM, CNN-BiLSTM, and TCN-BiLSTM.

In this paper, the overall performances of the LSTM, BiLSTM, CNN-BiLSTM, and TCN-BiLSTM models in predicting the RUL of IGBT modules are demonstrated, and the prediction results as shown in Figure 7. From Figure 7a, it can be seen that the overall performances of the BiLSTM, CNN-BiLSTM, and TCN-BiLSTM models are superior to that of the LSTM model alone. This is because the three models can better capture past and future contextual information through bidirectional encoding, overcoming the contextual limitations of unidirectional LSTM. In addition, it can be seen from the second half of Figure 7b that the trend of LSTM predictions diverges from the real data, indicating that it is difficult to accurately predict the engine voltage value using LSTM model due to its unidirectional information processing. Finally, the CNN-BiLSTM and TCN-BiLSTM slightly outperform BiLSTM, as these two models include additional feature extraction layers before the BiLSTM network, a thereby extracting more comprehensive features and further improving prediction accuracy. In summary, compared with existing technologies, CNN-BiLSTM and TCN-BiLSTM can better capture the contextual features of voltage values and accurately predict the future.

To quantitatively analyze the performance differences among the four models, we calculated the MAE, MSE, RMSE, and $r^{2}$ for these models during model training and optimization. The results are summarized in Table 5.

As shown in Table 5, the CNN-BiLSTM model outperforms the other models, achieving the lowest MAE, MSE and RMSE among all models, indicating the smallest and most stable prediction errors. Moreove, the $r^{2}$ of the CNN-BiLSTM model is higher than that of the other three models, demonstrating the strongest explanatory power and best fit for the target variable. Furthermore, the performance difference between CNN-BiLSTM and TCN-BiLSTM indicates that CNN component more effectively captures contextual features in voltage sequences, thus enhancing prediction accuracy.

**Figure 8:**
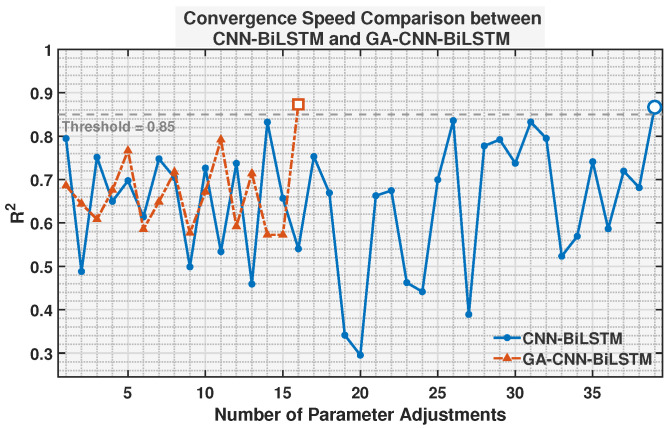
Comparison of the convergence speed between the GA-CNN-BiLSTM and CNN-BiLSTM models.

To verify the effect of the genetic algorithm on accelerating convergence, we compare the convergence speed between CNN-BiLSTM and GA-CNN-BiLSTM. The genetic algorithm is used to optimize the parameters of four models’ hyperparameters (number of convolution kernels, initial learning rate, regularization coefficient and number of LSTM hidden neurons), in which the lower limit of the hyperparameter search range is set to [50, 0.001, 1 × 10^−5^, 100] and the upper limit is set to [300, 0.01, 1 × 10^−3^, 300]. The core configuration of genetic algorithm is: population size of 10, maximum iteration times of 15, elite retention number of 2, crossover probability of 0.8. Adaptive feasible mutation function is adopted, real-time information is displayed and optimal fitness curve is drawn during iteration. The overall configuration balances optimization efficiency and search accuracy, which is used for efficient optimization of model hyperparameters. It should be noted that extensive experiments in this study have shown that when $r^{2}$ is greater than 0.85, the model can predict the RUL of IGBT modules with high accuracy. The experimental results are presented in Figure 8.

As shown in Figure 8, the GA-CNN-BiLSTM model can achieve optimal model parameters with fewer training iterations. Specifically, compared to the CNN-BiLSTM model, which requires 39 training iterations to achieve optimal parameters, the inclusion of the genetic algorithm allows GA-CNN-BiLSTM to gradually optimize parameters at the 5th, 10th, and 20th iterations. It ultimately reaches the optimal parameter values by approximately the 20th iteration. This indicates that the genetic algorithm can significantly accelerate the parameter optimization process, thereby improving training efficiency.

**Figure 9:**
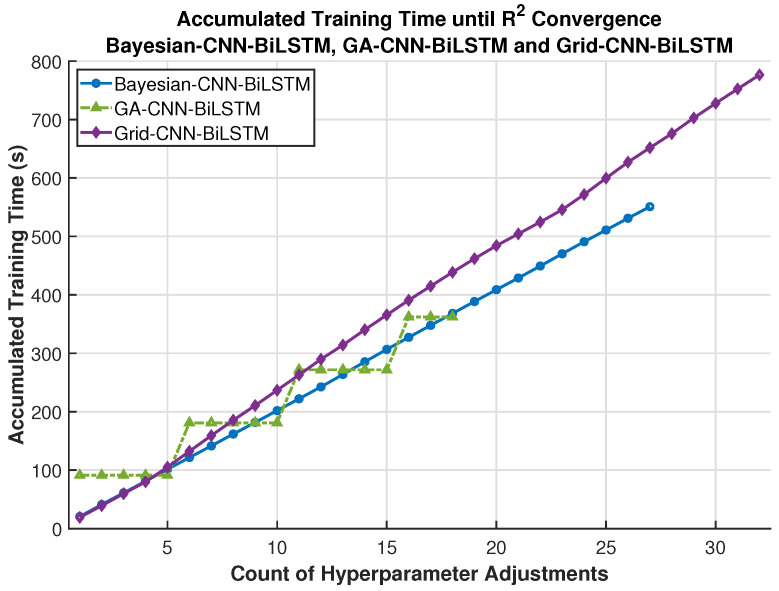
The performance of the model GA-CNN-BiLSTM, Bayesian-CNN-BiLSTM, and Grid-CNN-BiLSTM in terms of accumulated training time.

**Figure 10:**
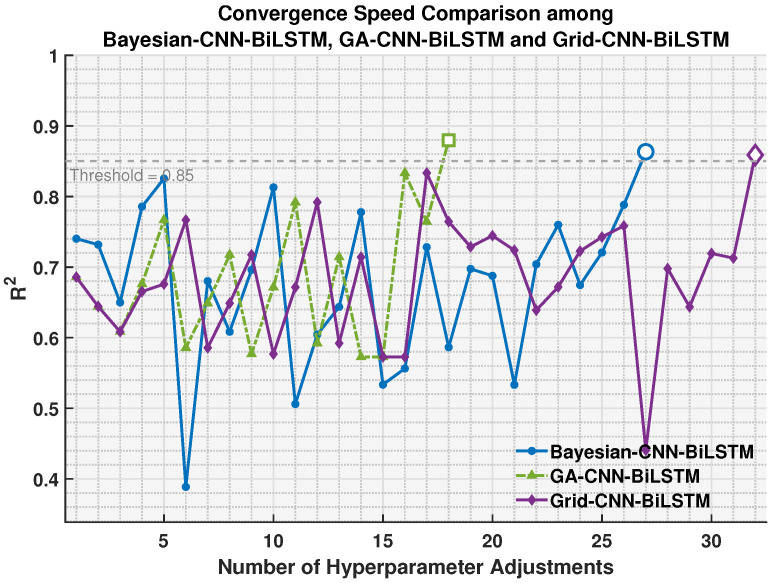
The performance of the models GA-CNN-BiLSTM, Bayesian-CNN-BiLSTM, and Grid-CNN-BiLSTM in terms of convergence speed.

Further experiments compare the time efficiency of three hyperparameter optimization algorithms: the proposed Genetic Algorithm (GA), the Bayesian algorithm, and the grid search algorithm. Figure 9 presents the accumulated training time required by the three algorithms to complete the training process. It is clear that GA-CNN-BiLSTM requires less total training time than Bayesian-CNN-BiLSTM and Grid-CNN-BiLSTM. This is because GA-CNN-BiLSTM can guide the optimization process to promising regions of the hyperparameter space, thereby reducing the number of ineffective hyperparameter trials. Meanwhile, it can also be observed from Figure 9 that when the number of hyperparameter adjustments is 5, all three models require nearly 100 s to complete 5 steps of training, indicating that the dominant computational cost stems from the training of the neural networks rather than the hyperparameter search procedure. Figure 10 then shows that GA-CNN-BiLSTM requires fewer hyperparameter adjustments, which can be attributed to the GA optimization strategy.

The excellent prediction accuracy and efficient optimization performance of the GA-CNN-BiLSTM model are critical for its real-world deployment in sensor networks. For IGBT modules, the core components in power supply units of sensor data acquisition nodes and actuator drivers, accurate RUL prediction enables predictive maintenance to prevent unexpected node failures, ensure data continuity, and maintain the overall health of the sensor network [24]. The model’s capacity to process multivariate time-series data (e.g., voltage, current, temperature) also fits well with the multi-sensor data streams common in networked monitoring systems.

## 5. Conclusions

To address aging-related failures of IGBT modules during long-term operation, this paper has proposed a RUL prediction method for IGBT modules based on a hybrid CNN and BiLSTM model optimized using a genetic algorithm. First, the failure mechanisms of IGBTs are analyzed to provide a theoretical basis for model development. Then, by leveraging the capability of CNNs to extract spatial features and the strength of BiLSTMs in capturing long-term temporal dependencies in time-series data, a CNN-BiLSTM-based RUL prediction model is constructed. Key hyperparameters are automatically optimized through a genetic algorithm to enhance convergence and training efficiency. Simulation results have demonstrated that the CNN-BiLSTM model outperforms baseline methods. These results confirm the effectiveness of the proposed approach for accurate RUL prediction of IGBT modules, while the genetic algorithm significantly improves the efficiency of the model optimization process.

## 6. Future Work

Future research will focus on advancing the engineering practicality and industrial application value of the proposed framework, with particular emphasis on its integration and deployment in sensor network systems [25]. First, we will incorporate high-fidelity, real-time monitoring data from distributed multi-sensor networks to further enhance the model’s adaptability and generalization performance under complex, time-varying operating conditions. Second, we will explore state-of-the-art optimization algorithms (e.g., particle swarm optimization (PSO), differential evolution), which will be adopted either as algorithmic performance benchmarks or embedded into a hybrid optimization framework to improve the model’s computational efficiency and prediction robustness. Finally, we will extend the proposed methodology from component-level IGBT RUL prediction to a system-level PHM architecture for full-stack sensor–actuator systems and distributed sensor network clusters [26], to support holistic, end-to-end predictive maintenance for industrial cyber–physical systems.

## Figures and Tables

**Table 1:** The model configurations of CNN-BiLSTM.

Layer Number	Name	Type	Description
1	Input_layer	Sequence input	5 × 1 × 1 dimension
2	conv_layer	2D Convolution	200 3 × 1 convolutions
3	relu_layer	ReLU	ReLU
4	flatten_layer	Flatten	Flatten
5	BiLSTM_layer	BiLSTM	BiLSTM
6	fc_layer	Fully connected	120 fully connected
7	dropout_layer	Dropout	50% dropout
8	output_layer	Fully connected	1 fully connected

**Table 2:** Comparison of common hyperparameter optimization methods.

Optimization Method	Advantages	Disadvantages
Grid search	Simple and intuitive, covering all possible hyperparameter combinations	High computational cost, especially when there are many hyperparameters or each parameter has multiple values
Random search	High efficiency, especially in cases where there is interaction between hyperparameters, it may find better configurations more quickly	Due to randomness, the optimal solution may be missed
Bayesian optimization	Reduce the number of evaluations, suitable for handling continuous parameter spaces	High implementation complexity and computational cost, especially when dealing with large-scale problems
Automated machine learning (AutoML)	Highly automated, integrating multiple technologies to improve efficiency, and suitable for complex hyperparameter combination problems	Requires high computing resources and has a high usage threshold
Metaheuristic optimization algorithms	Fast convergence on continuous optimization problems, insensitivity to initial values, and favorable parallelizability	Prone to local optima, performs poorly on discrete hyperparameters, and has no strict theoretical convergence guarantee
Swarm intelligence optimization algorithms	Strong global search capability and high adaptability	High computational load and complex parameter tuning

**Table 3:** Parameters of the CNN-BiLSTM model.

Layer Number	Parameter Description	Value
1	Number of model optimizations	1000
2	Maximum number of iterations	100
3	Number of convolution kernels	200
4	Number of hidden units in the BiLSTM layer	200
5	Number of units in the fully connected layer	120
6	Initial learning rate	[0.001, 0.005]
7	Dropout rate	[0.1, 0.5]

**Table 4:** Parameters associated with neural network training.

Parameter	Value
Initial learning rate	[0.001, 0.005]
Number of epochs	80
Batch size	20
Learning decay rate	[0.1, 0.5]
Decay cycle	[10, 40]

**Table 5:** Comparison of model performance based on MAE, MSE, RMSE, and $r^{2}$.

Model	MAE	MSE	RMSE	$r^{2}$
LSTM	0.1424	0.0324	0.1801	−0.2243
BiLSTM	0.0266	0.0011	0.0344	0.8068
CNN-BiLSTM	0.0220	0.0007	0.0279	0.8725
TCN-BiLSTM	0.0228	0.0008	0.0293	0.8599

## Data Availability

The data used in this study are obtained from the publicly available NASA prognostics center of excellence (PCoE) data repository. Specifically, the dataset used in this work is the IGBT accelerated aging data set, which is publicly released by NASA and can be accessed through the following links: Repository: https://www.nasa.gov/intelligent-systems-division/discovery-and-systems%20health/pcoe/pcoe-data-set-repository/, accessed on 16 January 2026. Dataset download: https://phm-datasets.s3.amazonaws.com/NASA/8.+IGBT+Accelerated+Aging.zip, accessed on 16 January 2026.

## Abbreviations

IGBTs	Insulated Gate Bipolar Transistors
RUL	Remaining Useful Life
BiLSTM	Bidirectional long short-term memory
GATE	GRU-Augmented Time-Frequency Estimato
PCA	Principal Component Analysi
FFNN	Feedforward Neural Network
SO	Snake Optimizer
POF	Physics-Of-Failure
PDFs	Probability Density Functions
CNN	Convolutional Neural Network
RNN	Recurrent Neural Network
GA	Genetic Algorithm
TSP	Traveling Salesman Problem
MAE	Mean Absolute Erro
MSE	Mean Squared Erro
PSO	Particle Swarm Optimization
